# Postoperative morbidity risk factors after conservative surgery of hydatic cyst of the liver: a retrospective study of 151 hydatic cysts of the liver

**DOI:** 10.1186/s12893-022-01570-7

**Published:** 2022-03-30

**Authors:** Mossaab Ghannouchi, Hawas Rodayna, Mohamed Ben Khalifa, Karim Nacef, Moez Boudokhan

**Affiliations:** Department of General Surgery, University Hospital Tahar Sfar, 5100 Mahdia, Tunisia

**Keywords:** Bile leakage, Morbidity, Mortality, Postoperative complication, Risk factor, Surgical complications

## Abstract

**Background:**

The purpose of the present paper is to assess the morbidity specifics risk factors of hepatic hydatid cyst after conservative surgery.

**Methods:**

We conducted a retrospective study of 102 patients over a period of 13 years, from 2006 to 2019. We included all patients operated on hydatid cyst of the liver, complicated and uncomplicated, in the Department of General Surgery in Tahar Sfar hospital, Mahdia, Tunisia. We excluded patients who received an exclusive medical treatment and those who have other hydatic cyst localizations.

**Results:**

The cohort was composed of 102 patients with a total of 151 cysts operated on using conservative surgery, among them there was 75 women (73.5%) and 27 men (26.5%). The median age was 43, with extremes ranging from 12 to 88 years. The majority of patients (94.1%) were from rural areas. The cysts were uncomplicated in about half of the cases (48%), elsewhere complications such as compression of neighboring organs (25.5%), opening in the bile ducts (16.7%), infection (9.8%), and rupture in the peritoneum (2%) were found. Conservative surgery was the mainstay of treatment with an overall mortality rate of 1.9%. The overall morbidity rate was 22%: 14% specific morbidity and 8% non-specific morbidity. External biliary fistula was the most common postoperative complication (9%). The predictive factors of morbidity in univariate analysis were: preoperative hydatid cyst infection (P = 0.01), Compressive cysts (P = 0.05), preoperative fever and jaundice, (respectively P = 0.03 and P = 0.02), no one achieved statistical significance in the multivariate model.

**Conclusions:**

Preoperative hydatid cyst infection, compressive cysts and preoperative fever and jaundice could be predictor factors of morbidity after conservative surgery for liver hydatid cyst. They must be considered in the treatment and the surgical decision for patients with hydatid cyst.

## Introduction

Hydatid cyst, also called cystic echinococcosis, is a widespread anthropozoonosis caused by the presence and development of the larval form of dog’s taenia: Echinococcus Granulosus in humans, the accidental intermediate hosts.

Its geographical distribution is directly linked to human–dog–sheep contact [1]. It is predominant in grazing areas in developing countries, especially in the Mediterranean region and the Middle East [2]. It infects up to 5% of people in some countries [3]. In Tunisia, the hydatid cyst of the liver is a real public health problem.

Hydatid disease can affect all organs; however, the liver remains the most commonly affected site (70%) [2].

Cysts could be revealed by complication, such as opening in the biliary tree, other complication, such as parietal complication, are more rare [4].

Despite the often-benign nature of the disease and the evolution of medical and surgical therapeutic approach of hydatid cyst, it remains marked by several postoperative complications; in fact, postoperative morbidity and mortality due to this disease may exceed 50% in some studies and the most frequent complication is external biliary fistula [5–7].

The aim of our work was to report the morbidity risk factors after conservative surgery in order to improve the immediate post-operative evolution and avoid long-term recurrences.

## Methods

This is a retrospective and descriptive study over a 13-year period from 2006 to 2019, involving 102 patients. We reviewed retrospectively all patients’ medical records. We included all patients operated on hydatid cysts of the liver, whether complicated or uncomplicated, in the Department of Surgery at Tahar Sfar University Hospital. We excluded liver hydatid cysts associated with intra thoracic locations or patients who were treated only with medical treatment.

The assessment of the cysts characteristics was based on radiological data: ultrasound (Gharbi classification), computed tomography (CT), and on intraoperative findings. The disease was confirmed intraoperatively by identifying the presence of hydatid membranes.

To carry out this work, the following sources were used: patient’s medical records, operational report registers and the anesthesia charts.

The data used for our study are:

- Epidemiological data: sex, age, geographic origin, dogs contact and history of hydatid cyst.
- Clinical data: the symptoms and their onset time, clinical examination.
- Biological and radiological examinations.
- The received treatment.
- Postoperative evolution and follow-up:ý
  - Post-operative monitoring: the operated patients were monitored according to the elements of the sign and the aspect of drainage.
  - Specific and non-specific morbidity: postoperative morbidity was defined as the occurrence of a specific or non-specific complication during the first 30 postoperative days.
  - Mortality: postoperative mortality was defined by the occurrence of death during the first 30 days postoperatively.

The statistical analysis was performed using the SPSS.20 software.

For the Univariate analyses, the comparison of the means was carried out using Student’s t test or by its nonparametric alternative (Mann–Whitney test) depending on whether the variables follow a Gaussian distribution or not. Comparisons of means between different subgroups of participants were obtained using the ANOVA test or its nonparametric alternative (Kruskal–Wallis test).

For the qualitative variables, the comparison was made by the Chi 2 test (χ^2^) or Fisher’s exact test.

The variables included in the multivariate analysis model were those with a P < 0.20 and were analyzed using a backward stepwise logistic regression model. The variables with a P ≤ 0.05 still present at the last step are therefore the significant independent variables of the multivariate model.

## Results

Sex distribution was 75 women (73.5%) and 27 men (26.5%), the sex ratio (F|M) was 2.77. The median age was 43, with extremes ranging from 12 to 88 years. The majority of patients (94.1%) were from rural areas. Contact with dogs was noted for 93 patients (91.2%). History of previous Hydatid cyst was found in 20 patients.

The clinical expression lacks specificity. Leading symptoms were pain in the right hypochondrium (87.3%) and fever (25.5%).

In the majority of cases, the cysts were uncomplicated (48%), elsewhere complications such as compression of neighboring organs (25.5%), opening in the bile ducts (16.7%), infection (9.8%), and rupture in the peritoneum (2%) were found.

Ultrasound was the key diagnostic exam. A total of 151 cysts were revealed by ultrasound: 68 patients had only one hydatid cyst, 23 patients had two hydatid cysts and 11 patients had multiple hydatid cysts. The average size of the cyst was 6.62 cm ± 4.66. According to Gharbi’s classification, type III cyst was the most common type (58 cysts) followed by type I (37 cysts) then types IV and II (28 and 22 cysts respectively), finally the least common cyst was type V (6 cysts).

Compression of the neighboring organs was the most frequent complication involving 26 patients (25.5%): compression of the inferior vena cava in 6 cases, the pancreas in 5 cases, of the stomach in 2 cases and of the spleen in 1 case. In 13 cases, the location of the compression was not mentioned.

The fistulization with passage of hydatid material into the bile ducts represented the second most frequent complication, involving 17 patients (16.7%). The infection of hydatid cyst was noted in 9.8% cases (10 patients) and the rupture of the cyst in the peritoneum was observed only in 2% of the cases (2 patients) (Table 1).

**Table 1:** Hydatid cyst characteristics

	Characteristics	Number
Number of cyst	1 cyst	68
	2 cysts	23
	Multiple	11
Ultrasound classification	Type 1	37
	Type 2	22
	Type 3	58
	Type 4	28
	Type 5	6
Localization	Left liver	57
	Right liver	59
	Hepatic dome	53
Size (cm)	< 5	51
	5–10	61
	10–15	29
	15–20	10

CT scan was performed in 94 cases. The results confirmed those found in Ultrasound exploration:

- Localization: 51 cysts were in the left liver, 50 cysts in the right and 50 cysts in the hepatic dome.
- Cysts type: 43 cysts were of type III, 20 cysts of type I, 16 cysts of type IV, 13 cysts of type II and 4 cysts of type V. For the other patients the type of the cyst was not mentioned.
- Size: The average size was 6.24 ± 4.06 cm, with extremes ranging from 3 to 20 cm.
- MRI was exceptionally performed; in fact it was performed only for 1 patient for an uncertain diagnosis.

Conservative surgery was the mainstay of treatment in all cases.

Eighty-five patients had uncomplicated cysts, they had a simple unroofing. Omentoplasty was used only for 16 patients (14.8%). Different intraoperative procedures were associated with the conservative treatment. Simple cholecystectomy was performed in 8 patients (7.8%) having a simple gallstone. Cholecystectomy with T-tube placement was performed in 16 patients (15.7%). Peritoneal cyst excision was performed in 2 patients who had peritoneal hydatidosis in addition to the liver hydatic cyst.

Twelve patients had a small cysto-biliary fistula (1 to 2 mm in diameter): 5 patients had unroofing with direct suture; the others were treated with unroofing with external drainage of the residual cavity.

Five patients had complicated cysts with a large cysto-biliary fistula (diameter > 5 mm): 2 patients had a bipolar drainage, 2 patients had a transparieto-hepatic fistulization and 1 patient had an internal drainage.

Medical treatment was widely administered in our patients. In fact, 78.4% of patients received medical treatment with Albendazole. It was administered preoperatively and generally maintained for 3 months after surgery.

The overall mortality rate was 1.9% (n = 2). Two cases of death occurred: one patient had a cysto-colonic fistula and the other had a giant hydatic cyst. The overall morbidity rate was 22% (n = 22): 8% non-specific morbidity and 14% specific morbidity: External biliary fistula, observed in 9 patients was the most common postoperative complication, followed by infection of the residual cavity in two patients, wound infection in two patients and bleeding of the residual cavity in one patient (Table 2).

**Table 2:** Post-operative complications

	Type	Number of patients
Specific morbidity	External biliary fistula	9
	Infection of the residual cavity	2
	Wound infection	2
	Bleeding of the residual cavity	1
Non specific complications		8

The length of hospital stay was 8.5 ± 6.7 days with extreme ranging from 4 to 51 days.

In univariate analyses, preoperative hydatid cyst infection was a predictor factor of postoperative morbidity (P = 0.01). Compressive cysts were also a predictor factor of postoperative morbidity (P = 0.05). Patients who had presented preoperative fever and jaundice, developed significantly more postoperative complications (P = 0.03 and P = 0.02 respectively) (Table 3).

**Table 3:** Univariate analysis of predictive factors of morbidity

Risck factor	P	OR	95% IC
Age (years)			
< 40	0.07	0.41	[0.14–1.17]
≥ 40			
Gender	0.53	1.08	[0.37–3.16]
Female			
Male			
Cyst number	0.91		
1	0.38	0.68	[0.2–2.35]
2	0.31	1.7	[0.46–6.23]
≥ 3	0.65	0.79	[0.09–6.92]
Cyst size (cm)	0.36	0.7	[0.23–2.13]
≤ 10			
> 10			
Cyst localisation	0.55		
Hepatic Dome	0.73	1.2	[0.4–3.59]
Right liver	0.77	0.73	[0.21–2.46]
Left liver	0.89	1.07	[0.38–3]
Preoperative hydatid cyst infection	**0.01**	9.37	[2.11–41.48]
Compressive cysts	**0.05**	2.68	[0.97–7.38]
Opening in the biliary ducts	0.25	1.79	[0.22–61.11]
Jaundice	**0.02**	4.5	[1.28–15.77]
Fever	**0.03**	2.9	[1.04–8.06]

After multivariate logistic regression, no one achieved statistical significance.

## Discussion

Hydatidosis constitutes a real public health problem in the world due to its prevalence and its postoperative morbi-mortality. In fact, despite the often-benign nature of the disease, the surgical approach is marked by several complications [8].

Mortality due to this disease can reach 4.5% [9–11]. Many mortality risk factors have been reported in the literature. Being aged > 40 years old is a risk factor of mortality according to Daradkeh [10]. Mortality is higher in complicated forms of hydatic cyst, especially when it is infected or opened in the thorax or in biliary ducts: the larger the communication is, the higher the mortality is [12]. Having multiple cysts is also a risk factor of mortality, especially when the cysts are treated at the same time [12]. Although Radical surgical techniques is the preferable treatment modality for some authors [13, 14], the majority consider it as more aggressive with higher mortality rate [3, 15–17]. Two deaths (1.9%) occurred in our study population, one patient had a giant cyst, the second had an opened cyst in the colon.

Morbidity rate in our study was 22%. In the literature, it ranges from 17.53 to 53.8% [9–11, 18] (Table 4). Surgical morbidity is the main problem of the hydatic disease in endemic country [16]. In our study, specific morbidity was 14%.

**Table 4:** Morbidity rate in literature

Author	Year	Morbidity rate (%)
BLAIRON [11]	2000	17.53
DARADHEK [10]	2006	53.8
EL MALKI [9]	2008	20.8
SECCHI [18]	2009	39
Our study	2020	22

External biliary fistula is the most frequent complication after surgery; its rate varies from 4.7 to 58.67% [10, 15, 18–21] magnetic resonance cholangiopancreatography (MRCP) and endoscopic retrograde cholangiopancreatography (ERCP) are the gold standard diagnostic tests [22]. This complication can be prevented if the connection between the cyst and the biliary tract is recognized and treated [21].

In our study, it was the major complication with a rate of 9%.

Suppuration of the residual cavity is the second most frequent complication of hydatic cysts. It has to be investigated in case of an opened cyst in the biliary ducts or in case of postoperative fever or when the drainage is purulent or the content is necrotic [16].

According to the literature, risk factors for morbidity are not well established and results are conflicting [23]. According to many studies [9, 10, 23, 24], morbidity is higher among patients older than 40 years, this may be associated with the presence of co morbidities. In our study age was not as a risk factor for postoperative morbidity (P = 007). Male gender has also been reported as a risk factor [23, 25]. According to Kayaalp, male gender is a risk factor for external biliary fistula (Sex male: 40.9% vs. 10.4%, P = 0.038). In our study, no significant difference was found between the two genders (P = 0.53).

According to Demicran [24], Atli [26], El Nakeeb [27] and Kayaalp [23] cholestase is a predictive factor for postoperative morbidity because in most cases it is related to an opened cyst in the biliary tree. In patients with cholangitis due to cystobiliary fistula; fluid electrolyte resuscitation and antibiotic therapy should be started rapidly and minimally invasive decompression of the biliary tract (percutaneous or ERCP assisted) should be performed [28].

In our study, fever and jaundice were also morbidity predictive factors (P = 0.03), (P = 0.02). The same results were reported by many other study in the literature [9, 23, 24, 26, 27, 29].

Morbidity depends also on the size of the cyst, its localization and the pericyst [30] as well as the number of the cysts when multiple cysts are found. According to El Malki [9] a cyst size > 10 cm is a predictive factor of postoperative complications. For Topcu [31], a cyst > 10 cm has more postoperative biliary complications. For Reddy [29], patients who developed a cysto-biliary communication had a cyst size ranging from 6.4 to 10.8 cm. In our study it was not a predictive factor of postoperative morbidity (P = 0.36).

Multiplicity of cysts has been reported as a risk factor of postoperative morbidity [32] but in our study it was not a risk factor (P = 0.91). According to the study of Zaouche [33], cysts localized in the hepatic dome and those deeply localized are risk factors for morbidity and mortality (16.3% vs 7.6%, P = 0.0006). In fact, hepatic dome cysts and those of segment I are more likely to cause postoperative morbidity requiring longer hospital stays than cysts of the left lobe and anterior localization (P = 0.0007).

In a retrospective study of 672 patients, El Malki [9], found that hydatic cysts localized in the hepatic dome are a predictive risk factor for morbidity (P < 0.05). Kayaalp [23], in a prospective study, proved that morbidity depends on the cyst localization. In fact perihilairy cysts, those localized in segments I, IV and V are more prone to be opened in the biliary ducts, with a higher risk of postoperative biliary fistula. The suppuration of residual cavity and length of stay are more important too [34]. In our study hepatic dome cysts were not found to be a risk factor for morbidity (P = 0.55).

In a retrospective study of 191 patients, Demicran [24] found that type III cysts are less likely to complicate during the postoperative period compared with other type (P = 0.032).

According to Akcran [15], the younger the cyst is (type I and II), the more likely to be open in the peritoneal cavity it is; however if the cyst is old, it could be opened in the biliary ducts.

Morbidity depends also on the surgical techniques used. Conservative surgery has more postoperative morbidity [35–37], radical techniques are more aggressive and has more mortality [16].

Radical surgery has better results of residual cavity and, therefore, a lower risk of infection, less postoperative biliary fistula, less morbidity and a lower risk of recurrence [9, 16, 38]. Yet, it involves more difficulties to control hemostase leading to more intraabdominale collection and postoperative hemorrhage [39].

Conservative surgery is the preferred technique in endemic countries [40]; in fact, in our study conservative technique was the only technique used. It is associated with some problems, mainly the residual cavities, a higher risk of infection, postoperative biliary fistula and a higher risk of recurrence.

Many procedures were proposed to reduce morbidity after conservative procedures such as omental packing the residual cavity, Obliteration of the residual cavity by imbricating sutures from within or external drainage [3, 39]. Those procedures could reduce morbidity by 40% in some studies [41], but results were discordant and debate is always on.

Omental packing could reduce morbidity due to the physiologic characteristics of the momentum. In fact it reduces intra abdominal infection and postoperative biliary fistula [42].

Obliteration of the residual cavity could erase the residual cavity by closing it wall one against the other. It could help to reduce the consequences of kysto-biliairy fistula [43].

External drainage could reduce morbidity by avoiding liquid retention in the residual cavity and could be used for irrigation and aspiration in case of infection of the residual cavity [36, 44].

According to El Malki [9] Obliteration of the residual cavity associated with external drainage is better than Obliteration alone (P = 0023). Akgun [45], in a prospective study of 102 patients, found better results with Obliteration of the residual cavity compared with external drainage (7% vs 49.5%, P < 0.05).

Another retrospective study by Demirci et al. [46] (n = 260) morbidity rate was 65.8% for patients who had a drainage of the residual cavity (n = 173) and 10.8% for those who did not have drainage of the residual cavity (n = 87). According to the same author, external drainage significantly increases postoperative morbidity in uncomplicated cysts and must be avoided whenever possible. In the patient group in which the cystic cavity was not drained, septic complications were rare.

Dziri in his study mentioned that drainage was associated with a higher rate of postoperative complication but without statically significant differences [47].

In the retrospective study of 116 patients by Killic et al. [48], there was no significant differences in the rate of postoperative biliary fistula in the management of residual cavity by external drainage, omental packing in or Obliteration of the residual cavity. The same result was found by Demicran et al. [24].

## Conclusion

Hydatidosis is a benign parasitosis whose treatment can lead to serious complications. It is a public health problem in the world on account of its frequency and postoperative morbi-mortality. In Tunisia, as in all Mediterranean countries, hydatidosis is an endemic disease. Surgery is the treatment of choice in the management of this condition. Conservative surgery is most common in endemic countries, but its success is highly depending on the residual cavity which is responsible in the short term for significant specific morbidity and a source of recurrence. The aim of our work was to analyze the epidemiological, diagnostic and therapeutic characteristics of this parasitosis, to evaluate morbidity after conservative surgery and to highlight the predictive factors of morbidity in order to optimize surgical treatment and limit recurrence.

In the literature many studies concluded that the postoperative complications seem to be influenced by certain factors such as: the multiplicity of cysts, the size, the site and the evolutionary stage. In our series, cyst infection and compressive hydatid cysts were considered as predictors of postoperative morbidity (P 0.01 and 0.05, respectively). Preoperative fever and jaundice were also postoperative morbidity factors (P 0.03 and 0.02, respectively). A better understanding of these different predictive factors would allow the surgeon to choose the most appropriate technique to reduce morbidity and mortality.

The results of our series were generally satisfying given the low rate of morbidity. However, it is believed that a prospective study with a larger sample and including different types of treatments could draw more solid conclusions.

## Data Availability

The datasets used and/or analyzed during the current study are available from the corresponding author on reasonable request.

## Abbreviations

CT: Computed tomography
MRI: Magnetic resonance imaging

## References

[1] Zait HAI, Guerchani MK, Hamrioui B (2013). Profil épidémiologique de 290 cas d’échinococcose kystique humaine diagnostiqués au CHU Mustapha d’Alger (2006 à 2011). Pathol Biol.

[2] Agudelo Higuita NI, Brunetti E, McCloskey C (2016). Cystic Echinococcosis. J Clin Microbiol.

[3] Klotz F, Nicolas X, Debonne J, Garcia J, Andreu J (2000). Kystes hydatiques du foie Encycl Med Chir (Edition Scientifiques et Medicales Elsevier SAS, Paris). Hepathologie.

[4] Akbulut S (2018). Parietal complication of the hydatid disease: comprehensive literature review. Medicine (Baltimore).

[5] Ennabli K, Gharbi S (1981). Les kystes hydatiques du foie ouverts dans les voies biliaires. A propos de 78 cas opérés au CHU de SOUSSE. Magreb Inf Med.

[6] Dziri CHK, Fingerhut A (2004). Treatment of hydatid cyst of the liver: where is the evidence?. World J Surg.

[7] Gourgiotis SSC, Moustafellos P (2007). Surgical techniques and treatment for hepatic hydatid cysts. Surg Today.

[8] Mormeche JSW, Sehili S, Khelifi S, Chammekhi CH, Baccar A (2009). Imagerie du KHF rompu dans les voies biliaires: a propos de 100 cas. J Radiol.

[9] El Malki HO, Mejdoubi Y, Souadka A, Mohsine R, Ifrine L, Abouqal R (2010). Predictive factors of deep abdominal complications after hydatid cysts of the liver: 15 years of experience with 672 patients reply. J Am Coll Surg.

[10] Daradkeh S, El-Muhtaseb H, Farah G, Sroujieh AS, Abu-Khalaf M (2007). Predictors of morbidity and mortality in the surgical management of hydatid cyst of the liver. Langenbecks Arch Surg.

[11] Blairon LDF, Hadj Hamida RB, Delmée M (2000). Le kyste hydatique du foie. Approche clinique et thérapeutique. À propos de 97 cas opérés dans un chu de tunisie centrale. Med Mal Infect.

[12] Ennabili KSR (1983). La mortalité post-opératoire des kystes hydatiques du foie. Lyon Chirg.

[13] Baimakhanov ZKS, Serikuly E, Doskhanov M, Askeyev B, Baiguissova D, Skakbayev A, Sadykov C, Barlybay R, Seisembayev M, Baimakhanov B (2021). Radical versus conservative surgical management for liver hydatid cysts: a single-center prospective cohort study. JGH Open.

[14] Akbulut SSA, Sezgin A, Cakabay B, Dursun M, Satici O (2010). Radical vs conservative surgery for hydatid liver cysts: experience from single center. World J Gastroenterol.

[15] Akcan ASE, Akyildiz H, Ozturk A, Atalay A, Yilmaz Z (2010). Predisposing factors and surgical outcome of complicated liver hydatid cysts. World J Gastroenterol.

[16] Noomen FMA, Fodha M, Boudokhane M, Hamdi A, Fodha M. Traitement chirurgical des kystes hydatiques du foie EMC. 2013.

[17] Sozuer E, Akyuz M, Akbulut S (2014). Open surgery for hepatic hydatid disease. Int Surg.

[18] Secchi MA, Pettinari R, Mercapide C, Bracco R, Castilla C, Cassone E (2009). Surgical management of liver hydatidosis: a multicentre series of 1412 patients. Liver Int.

[19] Kayaalp C, Senugal N, Akoglu M (2002). Importance of cyst content in hydatid liver surgery. Arch Surg.

[20] Julien C, Le Treut YP, Bourgouin S (2021). Closed cyst resection for liver hydatid disease: a new standard. J Gastrointest Surg.

[21] Yilmaz M, Akbulut S, Kahraman A, Yilmaz S (2012). Liver hydatid cyst rupture into the peritoneal cavity after abdominal trauma: case report and literature review. Int Surg.

[22] Akbulut S, Koc C, Sahin TT (2020). Comment on A serious complication of liver hydatid cysts in children: cystobiliary fistulas. Pediatr Surg Int.

[23] Kayaalp C, Bzeizi K, Demirbag AE, Akoglu M (2002). Biliary complications after hydatid liver surgery: incidence and risk factors. J Gastrointest Surg.

[24] Demircan O, Baymus M, Seydaoglu G, Akinoglu A, Sakman G (2006). Occult cystobiliary communication presenting as postoperative biliary leakage after hydatid liver surgery: are there significant preoperative clinical predictors?. Can J Surg.

[25] Ziser A, Plevak D, Wiesner RH, Rakela J, Offord KP, Brown DL (1999). Morbidity and mortality in cirrhotic patients undergoing anesthesia and surgery. Anesthesiology.

[26] Atli M, Kama N, Yuksek YN, Doganay M, Gozalan U, Kologlu M (2001). Intrabiliary rupture of a hepatic hydatid cyst: associated clinical factors and proper management. Arch Surg.

[27] El Nakeeb A, Salem A, El Sorogy M, Mahdy Y, Abd Ellatif M, Moneer A (2017). Cystobiliary communication in hepatic hydatid cyst: predictors and outcome. Turk J Gastroenterol.

[28] Akbulut S, Sahin TT (2021). Comment on the management of liver hydatid cyst with cystobiliary communication and acute cholangitis: a 27-year experience. Eur J Trauma Emerg Surg.

[29] Reddy AD, Thota A (2018). Cysto-biliary communication (CBC) in hepatic hydatidosis: predictors, management and outcome. Int Surg J.

[30] Sakhri J, Ali AB (2004). Le kyste hydatique du foie. J Chir.

[31] Topcu O, Sumer Z, Tuncer E, Aydin C, Koyuncu A (2009). Efficacy of chlorhexidine gluconate during surgery for hydatid cyst. World J Surg.

[32] Mounen M, Alaoui M, Fares FE, Mokhtari E. Les kystes hydatiques du foie a propos de 670 cas dont 552 compliqués Medécine du maghreb 1992:34.

[33] Zaouche A, Haouet K, Jouini M, El Hachaichi A, Dziri C (2001). Management of liver hydatid cysts with a large biliocystic fistula: multicenter retrospective study Tunisian Surgical Association. World J Surg.

[34] Beyrouti MI, Beyrouti R, Bouassida M, Ben Amar M, Frikha F, Ben Salah K (2007). Hydatid cysts of the spigelian lobe (segment I) of the liver: clinical and therapeutic particularities. Presse Med.

[35] Wejih D, Ramzi N, Karim A, Chadli D (2017). Le kyste hydatique du foie. Revue Francophone des Laboratoires.

[36] Cirenei A, Bertoldi I (2001). Evolution of surgery for liver hydatidosis from 1950 to today: analysis of a personal experience. World J Surg.

[37] Sayek I, Onat D (2001). Diagnosis and treatment of uncomplicated hydatid cyst of the liver. World J Surg.

[38] Yüksel O, Akyürek N, Sahin T, Salman B, Azili C, Bostanci H (2008). Efficacy of radical surgery in preventing early local recurrence and cavity-related complications in hydatic liver disease. J Gastrointest Surg.

[39] Safioleas MC, Misiakos E, Kouvaraki M, Stamatakos MK, Manti CP, Felekouras ES (2006). Hydatid disease of the liver: a continuing surgical problem. Arch Surg.

[40] Zaouche H, Haouet K. Traitement chirurgical des kystes hydatiques du foie EMC-Techniques chirurgicales-Appareil Digestif. 2006:1–17.

[41] A B. Kyste hydatique du foie. EMC Hépatologie 1993;A10(12p):7023.

[42] Wani AA, Rashid A, Laharwal AR (2013). External tube drainage or omentoplasty in the management of residual hepatic hydatid cyst cavity: a prospective randomized controlled study. Ger Med Sci.

[43] Gomez R, Moreno E, Colina F, Gonzalez I, Loinaz C, Garcia I (1995). Liver transplantation in patients with Budd-Chiari syndrome. Transplant Int.

[44] Hughet C, Nordlinger B, Zouache A, Quillichni M (1986). A Traitement chirurgical des kystes hydatiques du foie. Med Chir Dig.

[45] Akgun Y, Yilmaz G (2004). Efficiency of obliteration procedures in the surgical treatment of hydatid cyst of the liver. ANZ J Surg.

[46] Demirci S, Eraslan S, Anadol E, Bozatil L (1989). Comparison of the results of different surgical techniques in the management of hydatid cysts of the liver. World J Surg.

[47] Dziri C, Paquet J, Hay JM, Fingerhut A, Msika S (1999). Zeitoun G Omentoplasty in the prevention of deep abdominal complications after surgery for hydatid disease of the liver: a multicenter, prospective, randomized trial. J Am Coll Surg.

[48] Kilic M, Yoldas O, Koc M, Keskek M, Karakose N, Ertan T (2008). Can biliary-cyst communication be predicted before surgery for hepatic hydatid disease: does size matter?. Am J Surg.

